# Trombose induzida pelo calor endovenoso: relato de dois casos tratados com rivaroxabana e revisão da literatura

**DOI:** 10.1590/1677-5449.009816

**Published:** 2016

**Authors:** Walter Junior Boim de Araujo, Jorge Rufino Ribas Timi, Fabiano Luiz Erzinger, Filipe Carlos Caron

**Affiliations:** 1 Universidade Federal do Paraná – UFPR, Departamento de Cirurgia, Curitiba, PR, Brasil.

**Keywords:** varizes, laser endovenoso, ecografia vascular, trombose venosa

## Abstract

Define-se trombose induzida pelo calor endovenoso como a propagação do trombo a partir de uma veia superficial em direção a uma veia mais profunda. Em geral, é considerada clinicamente insignificante quando não há propagação do trombo para o sistema venoso profundo. Essa condição pode ser tratada com terapia anticoagulante, embora a observação pareça ser suficiente, principalmente para graus menores. Neste estudo, relatamos dois casos de trombose induzida pelo calor endovenoso que teriam indicação de heparina de baixo peso molecular até a resolução do quadro. Porém, optou-se pelo uso da rivaroxabana (15 mg de 12 em 12h), com resolução completa do trombo em 4 semanas (caso 1) e em 7 dias (caso 2). A rivaroxabana pode ser uma alternativa promissora no tratamento da trombose induzida pelo calor endovenoso avançada, pela simplicidade da posologia, sem comprometimento da eficácia ou da segurança. São necessários estudos prospectivos, randomizados e controlados que possibilitem melhor entendimento da condição e o desenvolvimento de recomendações mais definitivas sobre opções de prevenção e tratamento.

## INTRODUÇÃO

O mecanismo de ação da termoablação com laser endovenoso (em inglês, *endovenous laser treatment* – EVLT) no tratamento de varizes de membros inferiores é baseado na geração de calor que leva a dano endotelial, resultando assim no espessamento e na fibrose com oclusão não trombótica das veias incompetentes^1^.

A intensidade da contração da parede venosa parece ser importante, pois o lúmen residual da veia após o tratamento com laser é sujeito a oclusão pela formação de coágulos^2^.

A trombose induzida pelo calor endovenoso (em inglês, *endothermal heat-induced thrombosis* – EHIT) é definida como a propagação do trombo a partir de uma veia superficial em direção a uma veia mais profunda. Em geral, a EHIT é considerada clinicamente insignificante quando não há propagação do trombo para o sistema venoso profundo. Como tal, ela quase não é relatada na literatura, e a observação parece ser suficiente, principalmente para graus menores^3^.

Neste estudo, em uma análise retrospectiva das 278 safenas magnas e parvas tratadas através da técnica de EVLT em nosso serviço em um período de 5 anos, relatamos dois casos de EHIT em que se optou pela anticoagulação com rivaroxabana; pela simplificação da posologia, sem alteração da eficácia e da segurança, essa tem sido uma opção atraente na prevenção e no tratamento da trombose venosa profunda (TVP)^4^.

## DESCRIÇÃO DO CASO 1

**Figura 1:**
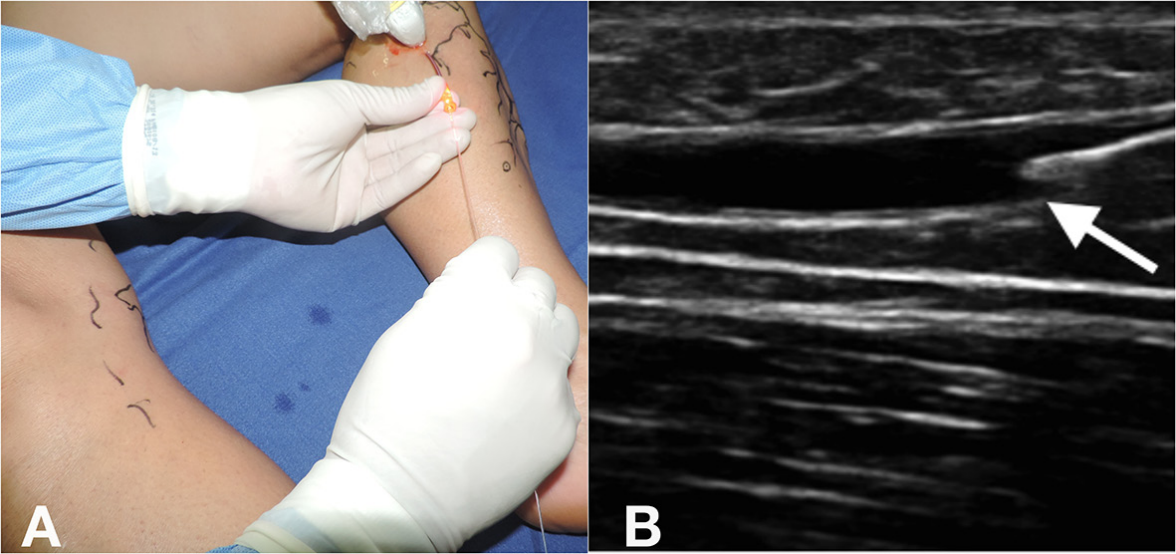
Punção ecoguiada (A) e passagem de fibra nua (*bare fiber*) (B).

Paciente do sexo feminino, 52 anos, diabética e hipertensa, com índice de massa corporal (IMC) de 31,2 e quadro clínico de varizes unilaterais de membro inferior esquerdo com classificação *Clinical-Etiology-Anatomy-Physiopathology* (CEAP) C5. Foi submetida a raquianestesia e colocada em decúbito dorsal. Posteriormente, foram realizadas punção ecoguiada e passagem de fibra nua (*bare fiber*) até 2 cm da junção safeno-femoral (JSF) (Figuras 1 e 2).

**Figura 2:**
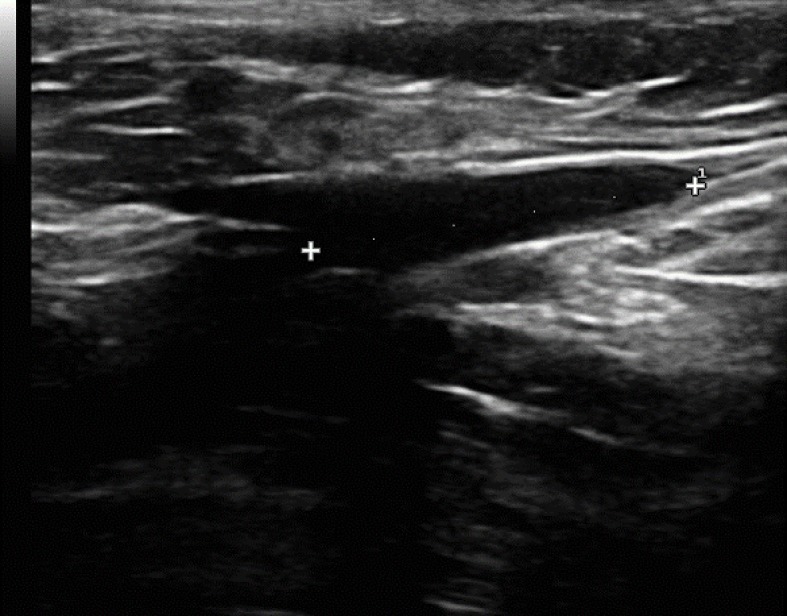
Posicionamento da fibra a 2 cm da junção safeno-femoral.

**Figura 3:**
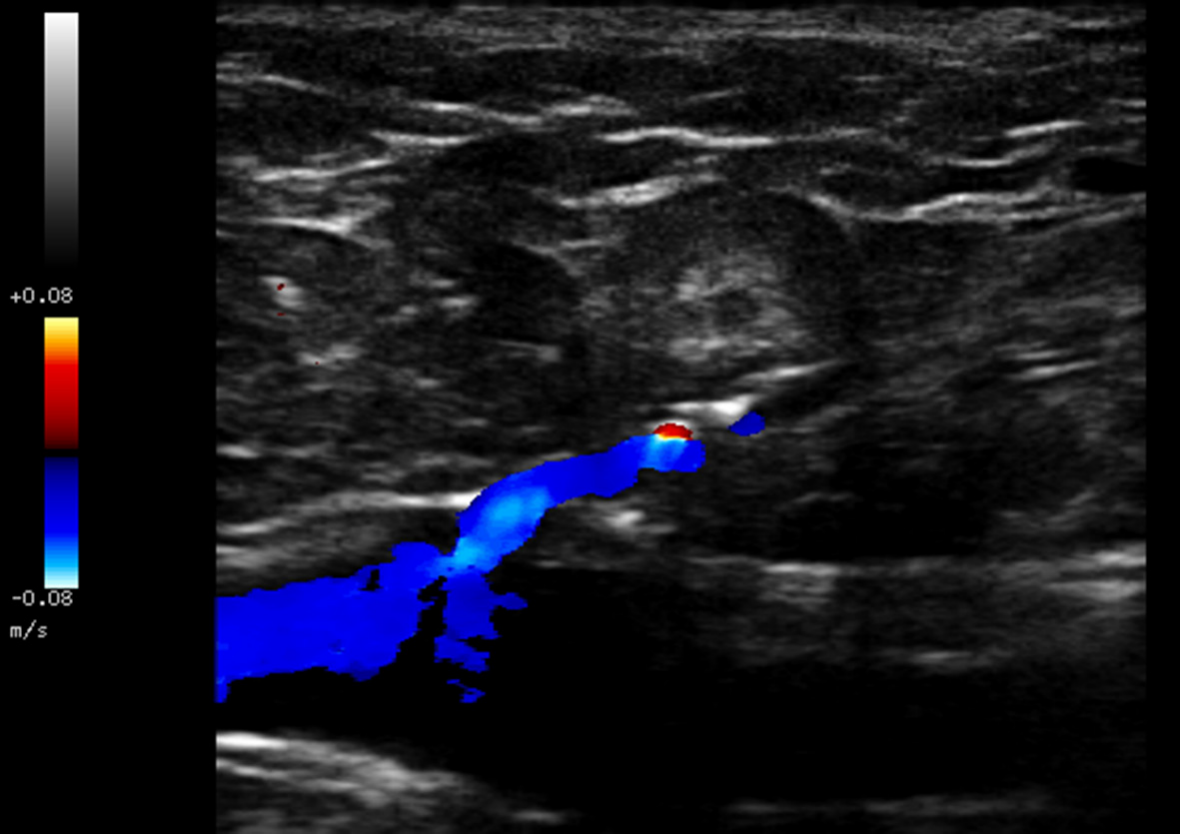
Controle imediato da junção safeno-femoral após termoablação.

Nesse momento, foi feita a tumescência com soro fisiológico em temperatura ambiente e efetuada a EVLT 1470 nm e a densidade de energia linear endovenosa (em inglês, *linear endovenous energy density* – LEED) de 38,5 J/cm, com bom controle ecográfico imediato da JSF (Figura 3).

Também foi realizada a retirada das varizes tributárias e das veias perfurantes-comunicantes insuficientes. A paciente recebeu dose única de enoxaparina de 40 mg 6 horas após o término da cirurgia; foi estimulada a deambulação a partir da recuperação anestésica. Recebeu alta após 10 horas do ato operatório, com prescrição de anti-inflamatórios e analgésicos.

Cinco dias após o procedimento, na primeira visita de acompanhamento, foi submetida a eco-Doppler colorido (EDC) de controle, que evidenciou a presença de EHIT com acometimento menor que 50% da luz da veia femoral comum esquerda; optou-se, então, pelo tratamento anticoagulante ambulatorial da paciente com rivaroxabana 15 mg de 12 em 12h. Repetido o exame em 4 semanas, foram evidenciadas a regressão do trombo e a resolução do quadro, momento em que o anticoagulante foi suspenso.

## DESCRIÇÃO DO CASO 2

Paciente do sexo feminino, 42 anos, com quadro de insuficiência venosa dos membros inferiores, classificação de CEAP C3, sem outras comorbidades e com história familiar (irmã) de TVP. Foi submetida a raquianestesia, punção de veia safena magna ao nível do joelho e EVLT 1470 nm, com utilização de fibra nua (*bare fiber*) com LEED de 36 J/cm na safena magna direita e de 42 J/cm na safena magna esquerda a partir de 2 cm da JSF, guiado por EDC, sem a realização de tumescência bilateral, sempre com paciente em posição de Trendelemburg. Associada ao procedimento, foi realizada a ressecção de varizes tributárias e perfurantes insuficientes nas pernas.

**Figura 4:**
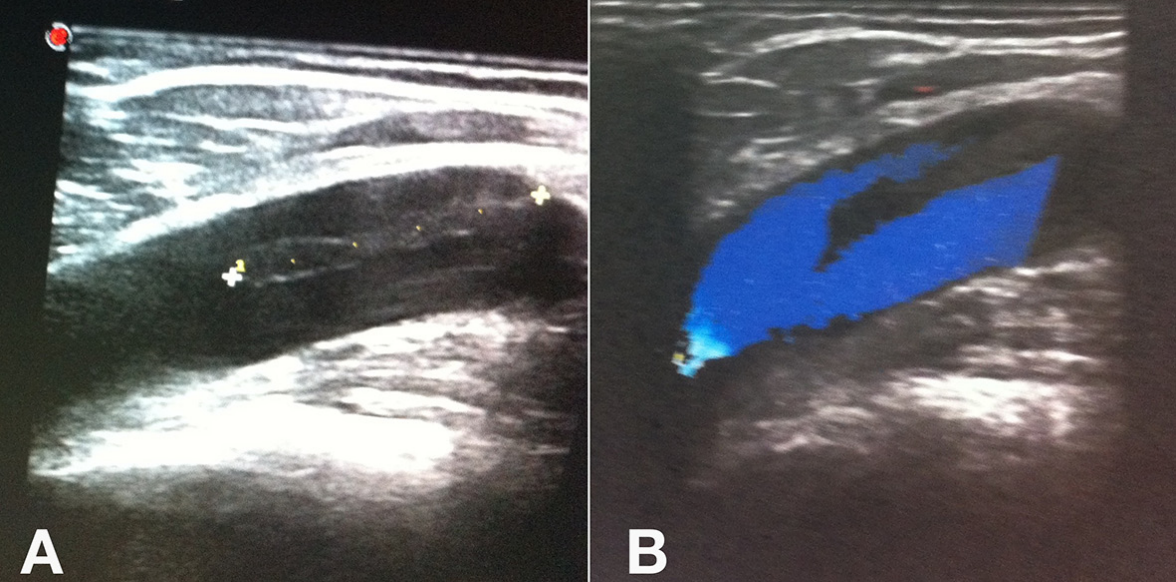
Trombose induzida pelo calor endovenoso com trombo de aspecto flutuante e progressão menor de 50% da luz na veia femoral comum. (A) Imagem ecográfica em modo B; (B) Imagem ecográfica em modo color.

**Figura 5:**
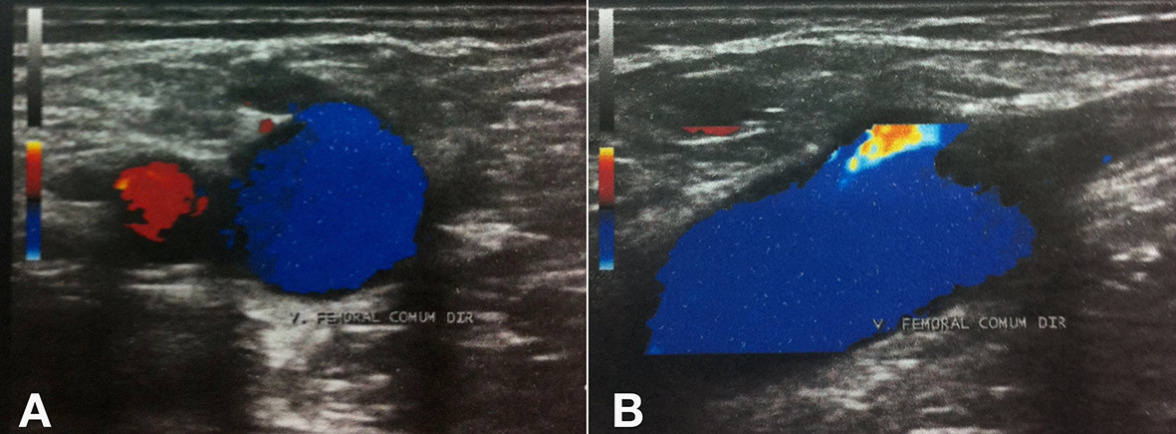
Eco-Doppler de controle após o sétimo dia de tratamento com rivaroxabana. (A) Imagem em modo color com transdutor na transversal; (B) Imagem em modo color com transdutor na longitudinal.

Recebeu alta no mesmo dia, com uso de anti-inflamatório e dose única de enoxaparina de 40 mg 6 horas após o término da cirurgia, sendo estimulada a deambulação diária. Mesmo orientada a retornar ao ambulatório no sétimo dia, a paciente compareceu somente no 28º dia de pós-operatório, quando foi submetida a EDC de controle, que evidenciou a presença de EHIT com trombo de aspecto flutuante e acometimento menor que 50% da luz da veia femoral comum direita (Figura 4). Foi realizado tratamento domiciliar com rivaroxabana 15 mg de 12 em 12h. No sétimo dia após a realização do EDC de controle, não mais se evidenciou a presença de trombo (Figura 5). A paciente permaneceu assintomática e completou três meses de anticoagulação. Teve os resultados de seus testes de trombofilia negativos, mas o desaparecimento do trombo após 7 dias chamou atenção para uma provável embolia pulmonar (EP). Por esse motivo, optou-se por realizar angiotomografia de tórax, que descartou essa hipótese diagnóstica.

## DISCUSSÃO

A EHIT é um resultado esperado no acompanhamento da EVLT da veia safena magna. O que ainda não está claro na literatura é a evolução clínica dos pacientes que apresentam EHIT nas proximidades ou que se estende para a JSF^5^.

Apesar de não existir evidência da correlação entre a distância do início da termoablação na JSF e a possibilidade de EHIT, pode ocorrer migração da fibra após a realização da tumescência, com deslocamento mínimo da fibra pela compressão externa^6^. Existe a sugestão de que a mudança da distância do início da termoablação em relação à JSF para 2,5 cm ou superior possa resultar em uma diminuição da incidência de EHIT^7^.

Diversos sistemas de classificação da EHIT foram desenvolvidos, podendo diferir no que diz respeito a necessidade, tipo de tratamento, dose e duração da anticoagulação^8^^-^ 10.

Kabnick et al.^8^ desenvolveram um sistema de classificação da EHIT de acordo com a extensão do trombo e sua propagação para o sistema venoso profundo (Quadro 1).

**Quadro 1:** Sistema de classificação para a trombose induzida pelo calor endovenoso descrito por Kabnick et al.^8^.

Classe	Extensão do trombo	Tratamento
I	Próximo à junção venosa superficial-profunda.	Conduta expectante, com seguimento seriado com ultrassom.
II	Prolonga-se para além da junção, com diâmetro de secção transversal de < 50%.	Heparina de baixo peso molecular até a resolução do trombo, com acompanhamento ecográfico.
III	Prolonga-se para além da junção, com diâmetro de secção transversal de > 50%.	Heparina de baixo peso e antivitamina K.
IV	Trombose venosa profunda totalmente oclusiva.	Heparina de baixo peso e antivitamina K.

Lawrence et al.^9^ também desenvolveram um sistema de classificação da extensão da termoablação endovenosa para a JSF, com uma proposta terapêutica para cada nível (Quadro 2).

**Quadro 2:** Sistema de classificação da extensão da termoablação endovenosa para a junção safeno-femoral descrito por Lawrence et al.^9^.

Classe	Extensão do trombo	Tratamento
1	Abaixo do nível da veia epigástrica superficial	Conduta expectante
2	No nível da veia epigástrica superficial	Conduta expectante
3	No nível da veia femoral comum	Escolha do cirurgião
4	Abaulando na veia femoral comum	Heparina de baixo peso
5	Após a junção safeno-femoral, adjacente à parede da veia femoral comum	Heparina de baixo peso
6	Na veia femoral comum, consistente com trombose venosa profunda	Heparina de baixo peso e antivitamina K

Harlander-Locke et al.^10^^,^ 11 descreveram um sistema de classificação da extensão da termoablação endovenosa para a junção safeno-poplítea, incluindo a incidência e uma proposta de tratamento para cada nível (Quadro 3).

**Quadro 3:** Sistema de classificação da extensão da termoablação endovenosa para a junção safeno-poplítea descrito por Harlander-Locke et al.^10^^,^ 11.

Nível	Extensão do trombo	Tratamento
A	> ou = 1 mm distal à veia poplítea	Conduta expectante
B	No nível da veia poplítea	Conduta expectante
C	Prolonga-se para além da veia poplítea	Escolha do cirurgião
D	Trombose venosa profunda totalmente oclusiva	Heparina de baixo peso e antivitamina K

Não há dados convincentes que embasem o uso rotineiro de anticoagulantes em dose profilática na EVLT. Os pacientes selecionados com história de trombose venosa superficial, TVP ou obesidade são candidatos à profilaxia^12^. Rhee et al.^6^, em uma análise retrospectiva de 519 procedimentos de EVLT ao longo de um período de 3 anos, encontraram o escore de avaliação de risco de TVP (escore de Caprini)^13^ e o sexo masculino como fatores de risco independentes. Concluíram que parece haver uma correlação entre trombofilia e EHIT, e que o cálculo de uma pontuação de avaliação de riscos para os indivíduos pode permitir uma previsão do aumento do risco para EHIT e indicar quais pacientes poderiam se beneficiar de profilaxia medicamentosa^6^.

Sufian et al. fizeram um estudo prospectivo para avaliar a incidência de EHIT e sua progressão, e concluíram que os fatores de risco incluem: tamanho da veia, idade e flebectomias concomitantes^14^.

A EP após procedimentos de EVLT é, felizmente, rara. Rosales-Velderrain et al. relaram três casos de pacientes que desenvolveram EP após a ablação por radiofrequência da veia safena magna e mini-flebectomias das varizes primárias sintomáticas dos membros inferiores^15^. Apesar dos estudos de profilaxia para TVP com uso de novos anticoagulantes estarem voltados para situações específicas, como as cirurgias ortopédicas^16^, o uso *off-label* destes na profilaxia das mais diversas situações clínicas e cirúrgicas tem sido uma prática cada vez mais frequente.

Atualmente, os anticoagulantes diretos dos fatores II (trombina) e Xa também têm sido utilizados para fins de anticoagulação, tornando-se uma opção simplificada para o tratamento da TVP^4^. O estudo EINSTEIN^17^ comparou o uso de rivaroxabana com o tratamento tradicional (enoxaparina-varfarina) da TVP e demonstrou não inferioridade de eficácia e segurança como monoterapia oral.

Werth et al.^18^ documentaram um caso de EHIT assintomática, classe I de Kabnick, que progrediu para classe III após 7 dias de conduta expectante e acompanhamento clínico. Após a utilização de rivaroxabana durante 14 dias, evoluiu para resolução completa do trombo.

Embora dados de apoio ainda sejam necessários, estamos de acordo com as diretrizes do Fórum Venoso Americano^19^, que recomenda a realização pós-operatória do EDC em 24 a 72h (grau de recomendação 2C), para reconhecer e tratar, principalmente, os pacientes com graus mais avançados de EHIT, com extensão do trombo na veia femoral.

No presente estudo, relatamos dois casos de EHIT classe II de Kabnick e nível 5 de Lawrence em que a indicação seria a utilização de heparina de baixo peso molecular (HBPM) até a resolução do quadro. Mesmo sabendo que o tratamento da EHIT em graus menores é mais controverso e deixado a critério do operador, queríamos tratar esses doentes com pelo menos um curso curto de anticoagulação. Devido ao mecanismo de ação semelhante, à facilidade do tratamento, à possibilidade de administração por via oral e à manutenção do tratamento em regime ambulatorial, optou-se pela utilização de rivaroxabana 15 mg de 12 em 12h, com resolução completa do trombo em 4 semanas no caso 1 e em 7 dias no caso 2.

## CONCLUSÃO

A rivaroxabana e os demais novos anticoagulantes orais podem ser alternativas promissoras para o tratamento dos casos avançados de EHIT, pela simplificação posológica. Com o aumento do número de procedimentos, a disseminação das técnicas de termoablação endovenosa e o aprofundamento do estudo das suas principais complicações, espera-se um maior número de documentações dos casos de EHIT e, consequentemente, a possibilidade de realização de estudos prospectivos, randomizados e controlados que possibilitem um maior entendimento e recomendações mais definitivas das opções de prevenção e tratamento.
