# Supplementary material for: Trombose induzida pelo calor endovenoso: relato de dois casos tratados com rivaroxabana e revisão da literatura
Source: J Vasc Bras. 2016 Apr-Jun;15(2):147–52. [Article in Portuguese] doi: 10.1590/1677-5449.009816 (PMC5829710; doi:10.1590/1677-5449.009816)
Supplement: Translation

# Translation: Endothermal heat-induced thrombosis (EHIT): reports on two case treated with rivaroxaban and literature review

**Authors:** Walter Junior Boim de Araujo, Jorge Rufino Ribas Timi, Fabiano Luiz Erzinger, Filipe Carlos Caron

**Affiliations:** 1 Universidade Federal do Paraná – UFPR, Departamento de Cirurgia, Curitiba, PR, Brazil.

**Keywords:** varicose veins, intravenous laser treatment, vascular ultrasonography, venous thrombosis

## Abstract

Endothermal heat-induced thrombosis (EHIT) is defined as propagation of a thrombus from a superficial vein into a deeper vein and is generally considered clinically insignificant if the thrombus does not propagate to the deep venous system. The condition can be treated with anticoagulation therapy, although monitoring may be sufficient, especially in less severe cases. In this paper we report on two cases of EHIT that met the criteria for indication of low molecular weight heparin until resolution of symptoms. However, the treatment actually chosen was 15 mg of rivaroxaban every 12 h and the outcomes were complete thrombus resolution in 4 weeks in Case 1 and 7 days in Case 2. Rivaroxaban may be a promising alternative for treatment of severe EHIT because the dosage regimen is simplified without compromising efficacy or safety. Prospective, randomized, controlled studies are needed to better understand EHIT and to develop more definitive recommendations on prevention and treatment options for this condition.

## INTRODUCTION

The mechanism of action of endovenous laser treatment (EVLT) for ablation of varicose veins in the lower limbs is based on generating heat that causes endothelial damage, resulting in thickening and fibrosis and culminating in non-thrombotic occlusion of the incompetent veins.^1^

The intensity of vein wall contraction appears to be important, because the residual vein lumen after laser treatment is subject to occlusion by clot formation.^2^ Endothermal heat-induced thrombosis (EHIT) is defined as propagation of a thrombus from a superficial vein into a deeper vein. It is generally considered clinically insignificant if the thrombus does not propagate to the deep venous system. As a result it is rarely described in the literature and observation appears to be sufficient, particularly for less severe cases.^3^

This paper describes two cases of EHIT from a retrospective analysis of 278 great and small saphenous veins treated with the EVLT technique at our service over a 5-year period. These two cases were treated using rivaroxaban for anticoagulation because it simplifies posology, without affecting efficacy or safety, making it an attractive option for prevention and treatment of deep venous thrombosis (DVT).^4^

## DESCRIPTION OF CASE 1

The patient was a 52-year-old female with diabetes and hypertension, a body mass index (BMI) of 31.2 and a clinical diagnosis of unilateral varicose veins of the left lower limb with a Clinical-Etiology-Anatomy-Physiopathology (CEAP) classification of C5. She was given spinal anesthesia and placed in decubitus dorsal. Next, ultrasound-guided puncture was performed and a bare optical fiber advanced up to 2 cm from the saphenopopliteal junction (SPJ) (Figures 1 and 2).

**Figure 1:** Ultrasound-guided puncture (A) and placement of bare fiber (B).

**Figure 2:** Fiber tip positioned 2 cm from the saphenopopliteal junction.

Tumescence was induced with saline solution at room temperature and then EVLT was conducted with a 1470 nm laser and linear intravenous energy density (LEED) of 38.5 J/cm, with a good control ultrasound of the SPJ immediately postoperatively (Figure 3).

**Figure 3:** Control scan of saphenopopliteal junction immediately after thermoablation.

During the same procedure, tributary varicose veins and incompetent perforating-communicating veins were removed. The patient was given a single 40 mg dose of enoxaparin 6 hours after the end of surgery and was encouraged to walk as soon as she had recovered from the anesthetic. She was discharged 10 hours after the operation, with a prescription for anti-inflammatories and analgesics.

Five days after the procedure, at the first follow-up appointment, the patient was examined with color Doppler ultrasonography (CDU), which detected an EHIT compromising less than 50% of the lumen and a decision was taken to choose outpatient anticoagulant treatment with 15 mg of rivaroxaban every 12h. The examination was repeated 4 weeks later and it was found that the thrombus had undergone regression and the clinical picture had normalized, which is when the anticoagulant was withdrawn.

## DESCRIPTION OF CASE 2

The patient was a 42-year-old female with venous insufficiency of the lower limbs and a CEAP classification of C3, free from other comorbidities, but with a family history (sister) of DVT. Spinal anesthesia was given and then the great saphenous vein was punctured at the level of the knee and EVLT was conducted with a bare fiber and a 1470 nm laser using a LEED of 36 J/cm for the right great saphenous vein and a LEED of 42 J/cm for the left great saphenous vein, starting 2 cm from the SPJ and guided by CDU, without inducing tumescence in either limb and with the patient in the Trendelenburg position throughout. During the same procedure, incompetent perforating veins and tributary varicose veins in the legs were resected.

The patient was discharged the same day on anti-inflammatories and a single 40 mg dose of enoxaparin 6 hours after the end of surgery and encouraged to walk every day. Despite having been instructed to return to the clinic 7 days later, the patient only returned 28 days after the operation, when she underwent a control CDU, which showed EHIT with a thrombus that appeared to be floating compromising less than 50% of the lumen of the right common femoral vein (Figure 4). She was prescribed home treatment with 15 mg of rivaroxaban every 12h. Seven days after the control CDU, the thrombus was no longer detectable (Figure 5). The patient remained asymptomatic and was kept on anticoagulation for 3 months. The results of thrombophilia tests were negative, but the thrombus having disappeared in 7 days was considered a warning sign of possible pulmonary embolism (PE) and so an angiotomography of the chest was conducted, which ruled out this diagnostic hypothesis.

**Figure 4:** Endovenous heat-induced thrombosis with thrombus with a floating appearance and extension of less than 50% of the lumen of the common femoral vein. (A) Mode B ultrasound image; (B) Color mode ultrasound image.

**Figure 5:** Control Doppler ultrasound after 7 days’ treatment with Rivaroxaban. (A) Color-mode image with transverse transducer; (B) Image in color mode with longitudinal transducer.

## DISCUSSION

Endothermal heat-induced thrombosis is a finding that is expected during follow-up after EVLT of the great saphenous vein. However, what is not entirely clear in the literature is the clinical progression of patients who exhibit EHIT close to or extending to the SPJ.^5^

Although there is no evidence of a correlation between the distance from the SPJ at which thermoablation is started and the likelihood of EHIT, it is possible that the fiber can migrate after tumescence is induced, with minimal displacement of the fiber by external compression.^6^ It has been suggested that increasing the distance from the SPJ of the thermoablation starting position to 2.5 cm or greater could result in reduced incidence of EHIT.^7^

Several systems for EHIT classification have been developed and they can differ in terms of the need for and type of treatment and anticoagulation dose and duration.^8^^-^ 10

Kabnick et al.^8^ developed a system for classifying EHIT according to the extent of the thrombus and its propagation into the deep vein system (Chart 1).

**Chart 1:** Classification system for endothermal heat-induced thrombosis described by Kabnick et al.^8^.

**Class**	**Extension of thrombosis**	**Treatment**
I	Up to the junction of superficial and deep venous systems.	Watchful waiting, with serial ultrasound follow-up.
II	Extends beyond the junction, with a cross-sectional diameter < 50%.	Low molecular weight heparin until resolution of the thrombus, with ultrasound follow-up.
III	Extends beyond the junction, with a cross-sectional diameter > 50%.	Low molecular weight heparin and vitamin k antagonist.
IV	Totally occlusive deep venous thrombosis.	Low molecular weight heparin and vitamin k antagonist.

Lawrence et al.^9^ also developed a system for classification of the level of endovenous thermoablation with relation to the SPJ, with a proposal for treatment for each level (Chart 2).

**Chart 2:** System for classification of extension of endovenous thermoablation to the saphenopopliteal junction described by Lawrence et al.^9^.

**Class**	**Extension of thrombosis**	**Treatment**
1	Below the level of the superficial epigastric vein	Watchful waiting
2	At the level of the superficial epigastric vein	Watchful waiting
3	At the level of the common femoral vein	Surgeon’s choice
4	Bulging into the common femoral vein	Low molecular weight heparin
5	Beyond the saphenopopliteal junction, adjacent to the wall of the common femoral vein	Low molecular weight heparin
6	In the common femoral vein, consistent with deep venous thrombosis	Low molecular weight heparin and vitamin k antagonist

Harlander-Locke et al.^10^^,^ 11 described a system for classification of extension of endovenous thermoablation with relation to the SPJ, including incidence and a proposal for treatment for each level (Chart 3).

**Chart 3:** System for classification of extension of endovenous thermoablation to the saphenopopliteal junction described by Harlander-Locke et al.^10^^,^ 11.

**Level**	**Extension of thrombosis**	**Treatment**
A	≥ 1 mm distal of the popliteal vein	Watchful waiting
B	At the level of the popliteal vein	Watchful waiting
C	Extends beyond the popliteal vein	Surgeon’s choice
D	Totally occlusive deep venous thrombosis	Low molecular weight heparin and vitamin k antagonist

There are no convincing data on which routine use of anticoagulants for EVLT at prophylactic doses could be based. Selected patients with a history of superficial venous thrombosis, DVT or obesity are candidates for prophylaxis.^12^ Rhee et al., conducted a retrospective analysis of 519 EVLT procedures over a 3-year period and found that DVT risk assessment score (the Caprini score)^13^ and male sex were independent risk factors. They concluded that there appeared to be a correlation between thrombophilia and EHIT and that calculating a risk assessment score for patients could enable prediction of increased risk of EHIT and indicate which patients could benefit from pharmaceutical prophylaxis.^6^

Sufian et al. conducted a prospective study to evaluate the incidence of EHIT and its progression and concluded that the risk factors include: size of the vein, age and concomitant phlebectomies.^14^

Thankfully, PE is rare after EVLT procedures. Rosales-Velderrain et al. reported on three cases of patients who developed PE after radio frequency ablation of the great saphenous vein and mini-phlebectomies of primary symptomatic varicose veins of the lower limbs.^15^ Although DVT prophylaxis studies with new anticoagulants are designed for specific situations, such as orthopedic surgery,^16^ off-label use of these drugs for prophylaxis is becoming more and more common in the most varied range of clinical and surgical situations.

Nowadays, drugs that work by direct inhibition of factor II (thrombin) and factor Xa are also being used for anticoagulation, offering a simplified treatment option for DVT.^4^ An EINSTEIN study^17^ compared use of rivaroxaban with traditional treatment (enoxaparin-warfarin) for DVT and demonstrated it was not inferior in terms of efficacy or safety when used for oral monotherapy.

Werth et al.^18^ documented a case of asymptomatic EHIT, class I on the Kabnick classification, that progressed to class III after 7 days of watchful waiting with clinical monitoring. After 14 days’ treatment with rivaroxaban the outcome was complete resolution of the thrombus.

Although supporting data are needed, we are in agreement with the American Venous Forum guidelines^19^ that recommend postoperative CDU at 24 to 72h (recommendation level of 2C) to identify and treat patients with more advanced degrees of EHIT, with extension of thrombus to the femoral vein.

In this paper we have described two cases of EHIT, Kabnick class II and Lawrence level 5, in which the standard indication would be low molecular weight heparin until resolution of the condition. Despite being aware that treatment of less severe EHIT is more controversial and left to the surgeon’s judgment, we wanted to treat these patients with at least one short course of anticoagulation. In view of the similar mechanism of action, the ease of treatment, the possibility of oral administration and of maintenance of treatment in an ambulatory setting, it was decided to use 15 mg of rivaroxaban every 12h, with complete resolution of the thrombus in 4 weeks in case 1 and in 7 days in case 2.

## CONCLUSIONS

Rivaroxaban and other new oral anticoagulants may be promising options for treatment of advanced cases of EHIT because of the posological simplification they offer. As the number of procedures increases, the techniques for endovenous thermoablation become more widespread and their principal complications are better studied, it is expected that more cases of EHIT will be documented, making it possible to conduct prospective, randomized and controlled studies that offer better understanding and lead to more definitive recommendations on the options for prevention and treatment.
